# First identification and molecular characterization of CTX-M-15 extended-spectrum β-lactamase and OXA-9 β-lactamase in *Haemophilus influenzae* in the Iberian Peninsula

**DOI:** 10.1128/aac.01649-25

**Published:** 2026-01-30

**Authors:** Aida González-Díaz, Miguel Pinto, Irene Cadenas-Jiménez, Sara Duarte, Carmen Ardanuy, M. Manuela Ribeiro, Sara Martí, Paula Bajanca-Lavado

**Affiliations:** 1 Microbiology Department, Hospital Universitari Bellvitge, IDIBELL-UB 16383, L'Hospitalet de Llobregat, Catalonia, Spain; 2 Research Network for Respiratory Diseases (CIBERES), ISCIII 38176, Madrid, Spain; 3 Genomics and Bioinformatics Unit, Department of Infectious Diseases, National Institute of Health Doutor Ricardo Jorge 59020, Lisbon, Portugal; 4 Department of Pathology and Experimental Therapeutics, University of Barcelona 16724, Barcelona, Spain; 5 Haemophilus influenzae Reference Laboratory, Department of Infectious Diseases, National Institute of Health Doutor Ricardo Jorge, Lisbon, Portugal; 6 Laboratório Microbiologia, Serviço Patologia Clínica ULS - São João, Porto, Portugal; University of Fribourg, Fribourg, Switzerland

**Keywords:** OXA-9, CTX-M-15, beta-lactamase, whole-genome sequencing, mobile elements, antimicrobial resistance, *Haemophilus influenzae*

## Abstract

We describe, for the first time, the presence and characterization of CTX-M-15 and OXA-9 in two *Haemophilus influenzae* strains: PTHi-14525 (CTX-M-15) from Portugal and HUB-HI042681 (OXA-9) from Spain. Multidrug-resistant PTHi-14525 carried *bla*_CTX-M-15_ (two copies) and *bla*_TEM-1_ in an ICE*HpaHUB5-like* element. HUB-HI042681, resistant to β-lactams and aminoglycosides, carried a novel ICE*HinHUB1*. These findings highlight the genomic plasticity and expanded resistome of *H. influenzae*, raising concerns about treatment and emphasizing the need for continuous genomic surveillance.

## INTRODUCTION

*Haemophilus influenzae* is a major cause of respiratory tract infections and invasive disease. Historically regarded as highly susceptible to antimicrobial agents, this view changed in the early 1970s with the first reports of ampicillin-resistant strains due to TEM-1 β-lactamase production (1–3). In the early 1980s, amino acid substitutions were described in the penicillin-binding protein 3 (PBP3), reducing β-lactam binding affinity through point mutations or genetic recombination, contributing to the stepwise resistance in *H. influenzae* to ampicillin and third-generation cephalosporins (4–7). The evolution of resistance in *H. influenzae* has been accelerated by horizontal gene transfer, through the acquisition of transposons and integrative and conjugative elements (ICEs) (8, 9). Of concern is the potential transfer of extended-spectrum β-lactamase (ESBL) genes from closely related species. The recent identification of the ESBL *bla*_CTX-M-15_, in a multidrug-resistant (MDR) *H. parainfluenzae* strain in Spain and France positions this species as a potential reservoir (10, 11). Given the natural competence of *H. influenzae*, the acquisition of such ESBL genes would represent a significant shift, layering potent enzymatic resistance onto existing mechanisms (12, 13). This could compromise the efficacy of current treatments for invasive *H. influenzae* infections, reinforcing the importance of monitoring interspecies transfer of resistance mechanisms to contain the dissemination of multidrug resistance (14, 15). The growing burden of β-lactam resistance has placed *H. influenzae* ampicillin-resistant on the WHO priority list for new antibiotics, a challenge further intensified by the emergence of strains resistant to three or more antibiotic classes (MDR) and to more than five classes (extensively drug-resistant). These strains are associated with ICEs, which facilitate the spread of resistance among genetically diverse species (16, 17).

Here, we report the first documented cases of acquired β-lactamase-mediated resistance involving *bla*_CTX-M-15_ and *bla*_OXA-9_ in *H. influenzae* detected in the Iberian Peninsula. The National Reference Laboratory for *Haemophilus influenzae* (NRLHi) at the Portuguese National Institute of Health coordinates nationwide surveillance of invasive *H. influenzae*. The first resistant isolate PTHi-14525, from Porto, was received at NRLHi for antimicrobial resistance confirmation and whole-genome sequencing (WGS) characterization. The second isolate HUB-HI042681 was obtained in the Hospital Universitari de Bellvitge (HUB), a tertiary hospital located in the Southern Barcelona area (Spain) and analyzed as a part of a surveillance program in Catalonia. Antimicrobial susceptibility was tested by broth microdilution using MICroSTREP plus panels (PTHi-14525) and STRHAE2 Sensititre panels (HUB-HI042681) (Table 1). Breakpoints were interpreted following European Committee on Antimicrobial Susceptibility Testing (EUCAST) guidelines (18).

**TABLE 1:** Antimicrobial resistance profiles of PTHi14525 and HUB-HI042681 isolates

	PTHi-14525	HUB-HI042681
Antimicrobial agent	MIC (mg/L)^*a*^	MIC (mg/L)^*a*^
Ampicillin	>8 (R)	>4 (R)
Amoxicillin/clavulanate	2 (S)	2/1 (S)
Cefotaxime	>8 (R)	≤0.06 (S)
Cefaclor	>16 (R)	–^*c*^
Cefuroxime	>8 (R)	≤0.5 (S)
Cefepime	0.5 (R)	≤0.25 (S)
Meropenem	0.5 (S)	≤0.25 (S)
Ciprofloxacin	≤0.06 (S)	≤0.03 (S)
Chloramphenicol	≤1 (S)	≤1 (S)
Tetracycline	≤1 (S)	≤1 (S)
Trimethoprim/sulfamethoxazole	>2 (R)	≤0.5/9.5 (S)
Azithromycin	>4 (R)^*b*^	1 (S)

^*a*^ Based on EUCAST breakpoints.

^*b*^ Based on ECOFF.

^*c*^ "–", Not tested.

Genomic DNA was extracted using either the QIAsymphony (PTHi-14525) or MagMax DNA Ultra 2.0 for KingFisher Flex (HUB-HI042681). For short-read sequencing, libraries were prepared using Nextera XT (PTHi-14525) and Illumina DNA Prep (HUB-HI042681), followed by paired-end sequencing on Illumina platforms. For long-read sequencing, libraries were prepared using the Native Barcoding Kit (SQK-NBD114.24) and sequenced on R10.4.1 flow cells (Oxford Nanopore Technologies). Bioinformatic analysis was conducted using Bactopia v3.0.0 (19), which includes hybrid assembly (Unicycler) (20), annotation (Prokka) (21), sequence typing (22), and antibiotic resistance scan (AMRFinder+) (23). Amino acid substitutions in PBP3, GyrA, ParC, DHFR, and DHPS were screened using Geneious R9. Species confirmation was conducted using rMLST (pubmlst.org/species-id). Assemblies were re-annotated using *H. influenzae* RdKW20 (L42023) as a reference, with a minimum identity threshold of 80%. Prophages were identified with PHASTEST (24), and only intact regions were analyzed. Mobile genetic elements harboring antibiotic resistance genes were aligned with Mauve against ICE*Hin1056* (AJ627386), ICE*Hin299* (AM884334), ICE*Hpa8f* (AM884335), ICE*HpaT3T1* (FQ312002: 1154165–1212168), ICE*HpaHUB1*–ICE*HpaHUB4* (25), and ICE*HpaHUB5* (26). Annotation of the novel ICE*HinHUB1* was refined by manually curating Prokka annotations, using Juhas et al. (9) as a reference in combination with BLASTx and UniProt searches. Representations were generated using the Geneious backbone, PHASTEST outputs, and CAGECAT (cagecat.bioinformatics.nl/). PTHi-14525 (ERR13337076, ERR15705790) and HUB-HI042681 (ERR15760486, ERR15760484) raw reads were uploaded to the European Nucleotide Archive and ICE*HinHUB1* was deposited at NCBI (PX634569).

Genomic analysis identified, for the first time, the presence of the ESBL enzyme CTX-M-15 in a Portuguese isolate (PTHi-14525) and the narrow-spectrum β-lactamase (NSBL) OXA-9 enzyme in a Spanish isolate (HUB-HI042681). The first case occurred in March 2024 and involved a 67-year-old male patient who was transferred from another hospital and admitted to the Intensive Care Unit of Unidade Local de Saúde de São João with community-acquired pneumonia complicated by pulmonary septic shock, in which *H. influenzae* was isolated from a bronchoalveolar lavage. The second case involved a 68-year-old female with a history of classical mycosis fungoides diagnosed in 2009. In March 2024, she presented to the emergency department with bacteremic pneumonia, and *H. influenzae* was isolated from both blood cultures and bronchoalveolar lavage. PTHi-14525 was resistant to ampicillin, cefotaxime, cefuroxime, cefepime, trimethoprim-sulfamethoxazole and azithromycin (Table 1). β-lactam resistance was explained by amino acid substitutions D350N, G490E, N526K, and A530S in the PBP3, the acquisition of *bla*_CTX-M-15_ (two gene copies) and *bla*_TEM-1_. HUB-HI042681 showed resistance to penicillin, ampicillin, amikacin, gentamicin, and tobramycin (Table 1) exhibiting PBP3 substitutions D350N, M377I, A502V, and N526K, and carried the *bla*_OXA-9_ and *bla*_TEM-1_ genes. PTHi-14525 was resistant to trimethoprim-sulfamethoxazole due to the I95L substitution in DHFR and an SFLYND insertion at position 65 of DHPS. Macrolide resistance was only observed in PTHi-14525 and was explained by the presence of *mef*(A) and *msr*(D). On the other hand, HUB-HI042681 carried the aminoglycoside resistance genes *aadA1*, *aac(6′)-Ib,* and *aph(3′)-Ia*. Both assemblies yielded complete genomes in a single contig.

**Fig 1:**
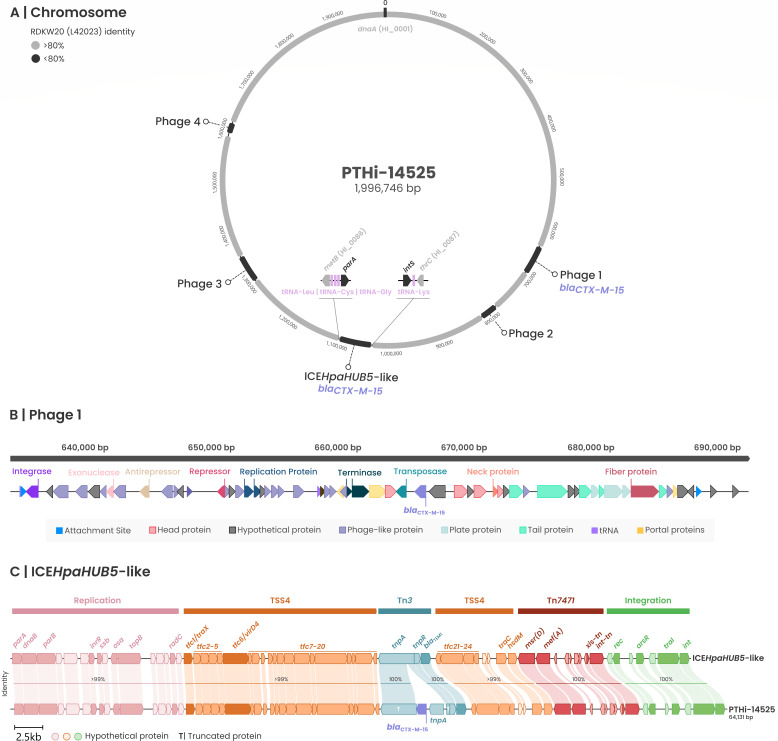
(**A**) Schematic representation of the PTHi-14525 chromosome. Regions sharing more than 80% sequence identity with *H. influenzae* Rd KW20 are shown in light gray. The four intact prophages detected by PHASTEST are indicated in dark gray, along with the ICE*HparHUB5-like* element and its chromosomal insertion site (gene names and Rd KW20 locus tag), tRNA (pink) and the first (*parA*) and last (*intS*) ICE genes. The *bla*_CTX-M-15_ genes are highlighted in purple. (**B**) Phage carrying the first copy of *bla*_CTX-M-15_. The genomic positions and gene types predicted by PHASTEST are shown. (**C**) Mauve alignment representation of the PTHi-14525 ICE elements carrying the second copy of the *bla*_CTX-M-15_ and the previously described ICE*HparHUB5-like* element, with pairwise sequence identity indicated for each region.

**Fig 2:**
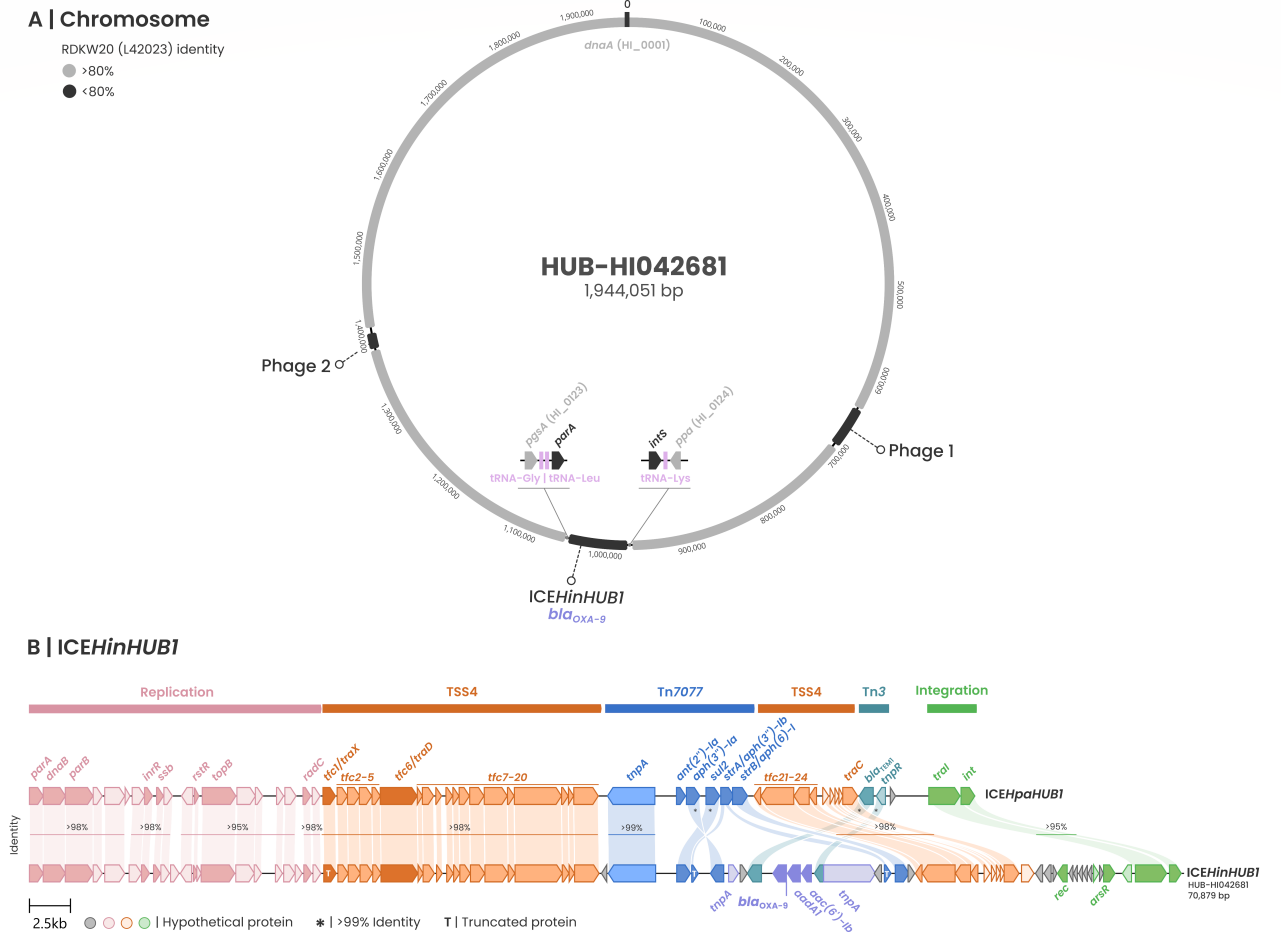
(**A**) Schematic representation of the HUB-HI042681 chromosome. Regions sharing more than 80% sequence identity with *H. influenzae* Rd KW20 are shown in light gray. The two intact prophages detected by PHASTEST are indicated in dark gray, along with the ICE*HinHUB1* element and its chromosomal insertion site (gene names and Rd KW20 locus tag), tRNA (pink), and the first (*parA*) and last (*intS*) ICE genes. The *bla*_OXA-9_ gene is highlighted in purple. (**B**) Mauve alignment representation of the new ICE*HinHUB1* elements carrying the *bla*_OXA-9_ and the previously described ICE*HparHUB1* element, with pairwise sequence identity indicated for each region.

The strains were nonencapsulated and showed 100% ribosomal MLST support for *H. influenzae*. Figure 1A depicts the chromosome of PTHi-14525 (ST2930), while Fig. 2A shows that of HUB-HI042681 (ST136). Four intact prophages were identified in PTHi-14525 using PHASTEST, and the predicted proteins are summarized in Supplementary Data set 1A. Predicted prophage 1 harbors the first copy of *bla*_CTX-M-15_, as shown in Fig. 1B. The second copy of *bla*_CTX-M-15_ was integrated together with a *tnpA* transposase within the Tn*3* transposon of a previously described ICE*HpaHUB5*-like element (identity >99%) (26), which also carries a Tn*7471* containing the *msr*(D) and *mef*(A) genes. HUB-HI042681 contained two prophages identified by PHASTEST and a novel ICE (ICE*HinHUB1*), carrying *bla*_OXA-9_, *bla*_TEM-1_, *aadA1*, and *aac(6′)-Ib*. The genetic structure of ICE*HinHUB1* is compared with its closest relative, ICE*HpaHUB1* (Fig. 2A). High identity was observed in the replication and T4SS genes, whereas significant differences were detected in the Tn*7077* region (Fig. 2B). The analysis of the genomic contexts revealed different arrangements of β-lactamase genes in the two studied isolates. PTHi-14525 harbored two copies of *bla*_CTX-M-15_, one integrated into a prophage and another within a Tn*3* transposon together with the *bla*_TEM-1_ gene, located in an ICE*HpaHUB5-like* element previously described (26). In contrast, the *bla*_OXA-9_ in HUB-HI042681 was identified in a novel ICE, designated ICE*HinHUB1*, in which transposons Tn*7077* and Tn*3* had merged into a new rearrangement. This new structure harbored multiple aminoglycoside resistance genes and the *bla*_TEM-1_ gene.

Recent studies have already identified the CTX-M-15 β-lactamase in *H. parainfluenzae* isolates from Spain and France (10, 11). While the urogenital origin of the CTX-M-15-positive *H. parainfluenzae* isolates precludes a direct ecological connection to respiratory *H. influenzae*, their existence demonstrates that this β-lactamase can be acquired by *Haemophilus* species. This establishes a precedent, suggesting that those *H. influenzae* isolated in the respiratory tract could similarly acquire the *bla*_CTX-M-15_ gene from other co-colonizing bacteria, reinforcing the role of such niches as potential reservoirs for resistance genes. The detection of *H. influenzae* isolates combining β-lactamases (either ESBL or NSBL), PBP3 amino acid substitutions, and additional resistance genes within mobile genetic elements underscores the species’ genomic adaptability to acquire and disseminate multiple resistance determinants (13–15, 27, 28). Both isolates likely acquired the resistance genes through horizontal transfer from co-colonizing species, facilitating the development of resistant strains capable of invading the bloodstream. Moreover, the patient carrying the β-lactamase CTX-M-15–producing isolate (PTHi-14525) had been transferred between at least three hospital units in two hospitals, suggesting a possible healthcare-associated acquisition and interspecies gene transfer from other hospital-associated pathogens (29, 30). This situation underscores the urgent need for ongoing surveillance, particularly through WGS, which allows the rapid detection of both novel and known resistance genes.

## SUPPLEMENTAL MATERIAL

Table S1 aac.01649-25-s0001.xlsx Intact phages detected by PHASTEST in both genomes.

ASM does not own the copyrights to Supplemental Material that may be linked to, or accessed through, an article. The authors have granted ASM a non-exclusive, world-wide license to publish the Supplemental Material files. Please contact the corresponding author directly for reuse.
